# Compliance with referral of sick children: a survey in five districts of Afghanistan

**DOI:** 10.1186/1471-2431-12-46

**Published:** 2012-04-27

**Authors:** William Newbrander, Paul Ickx, Robert Werner, Farooq Mujadidi

**Affiliations:** 1 BASICS/Afghanistan, Management Sciences for Health, Cambridge, USA; 2 Lawndale Christian Health Center, Chicago, USA; 3 UNICEF, Kabul, Afghanistan; 4 Management Sciences for Health, 784 Memorial Dr., Cambridge, MA, 02139, USA

**Keywords:** Referrals, Sick children, Integrated Management of Childhood Illness, Emergency pediatric care, Afghanistan

## Abstract

**Background:**

Recognition and referral of sick children to a facility where they can obtain appropriate treatment is critical for helping reduce child mortality. A well-functioning referral system and compliance by caretakers with referrals are essential. This paper examines referral patterns for sick children, and factors that influence caretakers’ compliance with referral of sick children to higher-level health facilities in Afghanistan.

**Methods:**

The study was conducted in 5 rural districts of 5 Afghan provinces using interviews with parents or caretakers in 492 randomly selected households with a child from 0 to 2 years old who had been sick within the previous 2 weeks with diarrhea, acute respiratory infection (ARI), or fever. Data collectors from local nongovernmental organizations used a questionnaire to assess compliance with a referral recommendation and identify barriers to compliance.

**Results:**

The number of referrals, 99 out of 492 cases, was reasonable. We found a high number of referrals by community health workers (CHWs), especially for ARI. Caretakers were more likely to comply with referral recommendations from community members (relative, friend, CHW, traditional healer) than with recommendations from health workers (at public clinics and hospitals or private clinics and pharmacies). Distance and transportation costs did not create barriers for most families of referred sick children. Although the average cost of transportation in a subsample of 75 cases was relatively high (US$11.28), most families (63%) who went to the referral site walked and hence paid nothing. Most caretakers (75%) complied with referral advice. Use of referral slips by health care providers was higher for urgent referrals, and receiving a referral slip significantly increased caretakers’ compliance with referral.

**Conclusions:**

Use of referral slips is important to increase compliance with referral recommendations in rural Afghanistan.

## Background

Child survival efforts in developing countries focus on applying basic lifesaving interventions to health problems faced by newborns, infants, and young children. These interventions are often applied by mothers or caretakers in the home, first-line health care providers such as community health workers (CHWs), or health care providers at the lowest-level health facility who have been trained to recognize common illnesses and provide basic treatment, such as oral rehydration solution and zinc for diarrhea. The importance for child survival of quick recognition and treatment of common child illnesses led to development of the Integrated Management of Childhood Illness (IMCI) approach by the World Health Organization and the United Nations Children’s Fund (UNICEF) in 1994.

A component of child survival that is less recognized and understood is the need for an effective referral system for infants and children who are very ill. A corollary requirement for a functioning referral system is caretakers’ compliance when a child is referred. If infants and children with severe illness that cannot be treated locally are either not referred or not taken to the next level of health facility, many of them will die of easily treatable conditions.

### The three key elements of referrals

A well-functioning referral system is one of the system components underpinning adequate implementation of IMCI. Three key elements of referrals underpin successful child survival efforts: (1) first-level health care providers must recognize when a child is very ill and needs to be referred as well as when a child does *not* need referral; (2) when referrals are appropriate, caretakers must comply with the referral for a very ill child to receive the intervention they require; and (3) higher-level health facilities must be ready to receive referrals and treat the children quickly and appropriately. All three elements of the referral system must function properly if child mortality is to be reduced.

### Research on referral systems in developing countries

Several studies from developing countries have addressed different aspects of referral systems. A study by Bossyns et al. [1] in Niger examined referral rates between health centers and a district hospital as well as parental and family compliance with referrals. It found that low referral rates and low compliance rates with referrals for young children were associated with increased child mortality. A retrospective study in Tanzania [2] concluded that too few children are referred, based on a combination of a low referral rate (0.6%) from primary health care facilities to higher levels, and a high admission rate (71%) at hospitals for children that were referred. The authors concluded that the findings highlighted a need for the adoption of the IMCI strategy in the more sparsely populated areas if child mortality rates were to be reduced.

A multi-country study found that lack of compliance with referrals can overburden first-level facilities with too many children who are very ill [3]. In Zimbabwe, self-referral by parents caused a different problem because parents could not distinguish among the types of health facilities to which their children were referred, resulting in an overburdening of referral centers with patients who could have been treated at a lower level. Excessive referral adversely affected the care of cases that were self-referred because they were not treated appropriately or in a timely manner due to overcrowding at these higher-level facilities [4].

Studies have scrutinized the use of IMCI guidelines by health care providers for providers’ competency in using them, appropriateness of referrals, cost efficiency, and correlation with various outcomes, in some cases resulting in modification of the algorithms used for determining when to refer sick children [5,6]. These studies concluded that the IMCI guidelines show good sensitivity for sepsis and pneumonia [7], and malaria [8], but in some cases lead to over-referral of cases that could have been treated at first-level health facilities [8]. The opposite problem, under-referral of cases, can have dramatic consequences for child survival. A study in Ghana found a 55% compliance with referrals of children; however, less than 1% of children were treated [9]. The authors estimated from health management information system data that nationally there were 169,425 “missed referrals” in that year, resulting in potentially thousands of children not surviving because they were not referred to receive appropriate treatment for their severe illness.

Research has reinforced our understanding of the importance of a properly functioning referral system as well as proper recognition of very ill children by first-level health care providers using IMCI guidelines to achieve maximum effectiveness of referrals. This led us to develop a guide for program managers to assess referral systems [10]. The third element of referral effectiveness, which hinges on compliance with referrals, however, has been relatively less studied. In Brazil, Alves da Cunha et al. [11] found just over one-half of families adhered to IMCI referrals of children to a higher-level health facility. A similar study in Sudan [12] showed only 44% compliance with referrals of very ill children. In both studies, many families claimed that the reason for low adherence with the referral was the improved condition of the child (35% in Brazil and 90% in the Sudan). Although this low adherence could be a result of over-referral, in both countries the data indicated that at least some of the sick children whose families did not comply with the referrals truly needed treatment at a higher level. The Sudan study found that better compliance with referrals was associated with the family caretaker’s level of education, with provision of medicines during the first visit, and with a short period between the first visit to the first-level health care provider and a follow-up visit to the same provider (probably meaning that the family recognized a deterioration in the child’s condition). In Ecuador, Kalter et al. [13] found that families who were given a referral slip and told to go immediately to the hospital were more likely to comply with referrals. In Uganda, a referral compliance of only 28% was in part explained by access barriers experienced by the family: financial limitations, transportation problems, and home responsibilities [14].

### Background on child survival and the referral system in Afghanistan

Child survival has been a priority of the Ministry of Public Health (MOPH) of Afghanistan since 2002 because of the high mortality rates of infants and children under 5 years of age. Afghanistan’s Basic Package of Health Services (BPHS) [15] was developed in 2003 to prioritize the interventions that would have the greatest impact on maternal, infant, and child mortality rates as well as on the diseases that cause the heaviest burden on the population. The BPHS included IMCI and other key child survival interventions. The BPHS recognized the importance of a well-functioning referral system: “[these priority primary health care interventions] would only work if a functioning hospital system existed that could accept referrals of complicated cases and conditions from health posts, basic health centers, and comprehensive health centers” [15]. There was a remarkable decrease in the mortality of children under age 5 and infant mortality in the 3 years after the introduction of the BPHS, from 2003 to 2006: the under-5 mortality rate decreased by 25%, from 257 to 192 per 1,000, and the infant mortality rate declined from 165 to 129 per 1,000 live births. Despite these significant reductions, Afghanistan’s under-5 and infant mortality rates remain among the highest in the world.

**Figure 1:**
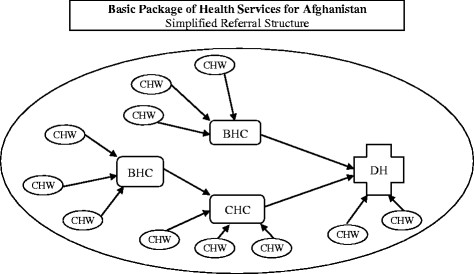
**Referral Paths for the Basic Package of Health Services**. Legend: CHW, community health worker; BHC, basic health center; CHC, comprehensive health center; DH, district hospital.

In theory, referral of sick children should go from the household to the CHW, and then to the different facilities: household to CHW to basic health center to comprehensive health center to district hospital. In reality, patient flow is more as illustrated in Figure 1, where CHWs can refer to different facilities, including the district hospital.

Information about referrals is lacking in Afghanistan. For example, data from the MOPH health management information system indicate that while 97% of health facilities have referral slips available, the median number of referrals represents only 1.6% of total encounters. While the MOPH has commissioned assessments of the knowledge of health issues and care-seeking behavior by patients and of health workers’ competency in making referrals [16], no further analysis has been undertaken to ascertain why so few patients in Afghanistan are referred, if there is a lack of compliance with referrals of sick children, and, if so, what the causes of noncompliance are. A rapid assessment of child and adolescent health by the MOPH and the Basic Support for Institutionalizing Child Survival (BASICS) Project in 2008 provided the first indication of a possible gap in the referral system: “The HMIS [health management information system] shows that far more patients are referred out from lower level facilities than registered as referred in at higher level facilities. While some of this may be due to under-reporting of referred-in patients, the trend is general enough to most likely reflect reality.”

In collaboration with the Child and Adolescent Health Directorate of the MOPH, BASICS conducted a household survey in February 2009 to gather data on 5 districts where an integrated child survival package was to be introduced. A portion of the survey was designed to answer questions about referral patterns in rural areas, such as parental compliance with referrals for sick children and barriers to compliance. This study aimed to understand issues with the functioning of the referral system for children in Afghanistan, a fragile state with a recently rebuilt public health system, and to identify factors that might influence referral compliance of sick children to higher-level health facilities.

## Methods

The 2009 baseline survey covered households in 5 rural districts in 5 provinces: Farza (Kabul Province), Shahfoladi (Bamyan Province), Ghorian (Herat Province), Farkhar (Takhar Province), and Qurqin (Jawzjan Province). These household surveys used the same sampling method as that of the annual household surveys of the MOPH’s USAID-funded Partnership Contracts for Health Services through nongovernmental organizations (NGOs) in those five provinces. The standard procedures for informing respondents of the purpose of the assessments and the guarantee for anonymity used in the annual household surveys were applied and the survey did not introduce new interventions, nor prevented access to interventions nor exposed individuals to possible harm.

As part of a lot quality assurance sampling (LQAS) method applied to sick children under 2 years of age in 5 districts, we first selected at least 130 households to be surveyed in each district, with the hope of yielding 100 households per district in which there had been a sick child within the previous 2 weeks. The planned total sample involved 100 households drawn from each of the 5 districts, for a total of 500. We used the listings of all the households in those 5 districts to identify the households belonging to 5 supervisory areas in each district. A supervisory area is a defined part of a district in which the NGO responsible for delivering health services and the MOPH regularly oversee all health activities. Within each supervisory area, at least 19 households were selected randomly.

In total, 492 children of 2 years of age or less who had been ill with acute respiratory infection (ARI), diarrhea, or fever within the previous 2 weeks were identified and included in the study. The parent or caretaker was interviewed only if there had been a sick child in the household within the previous 2 weeks. If there had been no sick child in any of the randomly selected households, the surveyor went to the nearest household seeking the presence of a sick child within the previous 2 weeks. The surveyor continued moving to the nearest household until a household with a sick child was identified in place of the initially randomly selected household. This is why more households were sampled than the intended sample of 100 households per district. In households in which a child under 2 years of age had been sick in the previous 2 weeks, the surveyor sought verbal consent from the household member for participating in the survey, as recommended in the procedures of the standard LQAS HHS in Afghanistan.

At each household where a sick child was identified, the surveyor used a structured questionnaire to ask the parent or caretaker a series of questions about the nature of their child’s illness; the nature of the illness; whom they had consulted outside the home for the illness; if they were referred, how they complied with a referral; and any real or perceived problems in accessing the next-level health facility that affected compliance with the referral, including geography, distance, transportation, and costs related to compliance with the referral. The data that were obtained differentiated between children who had been referred outside the home and those who had not been referred outside the home for their illness.

The survey fieldwork was carried out by data collectors and supervisors overseeing their sampling work. The surveyors were staff of the NGO providing services in the district. The staffs received 2 days of training and were checked to obtain more than 90% inter- and intra-surveyor reliability in using the survey questionnaires. Before leaving a household, the surveyor checked that all the questions had been completely answered. After a district was sampled, the survey supervisors ensured that all surveys were checked for completeness. If there were any missing responses, the surveyor would return to that household and complete the remaining questions. A second surveyor performed a 5% re-survey of the sampled households to check the reliability of the survey results. Upon completion of all the surveys, the data were reviewed for completeness and coded for entry into a database. When all the data were available, we held a workshop to analyze the data and review the results with the nongovernmental organizations and seek solutions to problems identified.

Statistical significance was tested by the two-tailed Fisher exact test for 2x2 contingency tables and the chi-square test for independence for larger contingency tables, using GraphPad InStat version 3.1, 32 bit for Windows, GraphPad Software, San Diego California USA, http://www.graphpad.com

## Results

For analysis, first sources of care were aggregated as follows, unless otherwise specified:

*CHW* = official public-sector community-level care, offering services according to the Basic Package of Health Services (BPHS). *BPHS facilities* = official public-sector facility-based care, including basic health centers and comprehensive health centers, often jointly referred to as “clinics”, and district hospitals, offering services according to BPHS. And *Other*s = private clinics and pharmacies, relatives and friends, and traditional healers, not necessarily offering services according to BPHS.

### Care-seeking for sick children by type of illness and source of care

Table 1 shows the trends in care-seeking behavior and causes of illness. From the sample of 492 sick children, 302, or 62%, were taken outside the home for advice on the child’s illness. The pattern of illnesses of the 492 children shows that over half suffered from ARI, while over 22% were ill from diarrhea and 22% from fever. There is a statistically significant relationship between the illness and seeking care outside the home (chi^2^: 12.479; p = .0020), with significantly more care-seeking outside the home for fever (74%) than for ARI (61%, p = .0131) or diarrhea (51%, p = .0005), but no statistically significant difference between ARI and diarrhea.

**Table 1:** Care-seeking outside the home, sources of care and type of health facility, by type of illness

**Sought care outside home**										
**Illness**	**No.**	**No**		**Yes**		**p-value**^**1**^				
ARI	274	108	39%	166	61%	.0131				
Diarrhea	110	54	49%	56	51%	.0005				
Fever	108	28	26%	80	74%					
Total	492	189	39%	302	61%					
1. Comparing ARI with Fever and Diarrhea with Fever										
First source of care outside home										
Illness	No.	Traditional healer		Relative/ friend		Private clinic or pharmacy		Public-sector facility		p-value^2^
ARI	274	2	1%	18	11%	29	18%	117	71%	.0001
Diarrhea	110	1	2%	10	18%	10	18%	35	63%	.0357
Fever	108	3	4%	24	30%	19	24%	34	43%	
Total	492	6	2%	52	17%	58	19%	186	62%	
2. Comparing Public-sector facility with all others combined										
Type of public-sector facility										
(when first source of care was a public-sector facility)										
Illness	No.	CHW		BPHS clinic		BPHS hospital				
ARI	117	30	26%	69	59%	18	15%			
Diarrhea	35	5	14%	20	57%	10	29%			
Fever	34	6	18%	21	62%	7	21%			
Total	186	41	22%	110	59%	35	19%			

Where were the 302 sick children taken when health care was sought? More than 3 of every 5 sick children (62%) who were taken outside the home to a health care provider went to a public-sector CHW or BPHS facility, to be treated by a CHW at a clinic or at a hospital (Table 1). There is a statistically significant relationship between the type of illness and whether care was sought from a CHW and in a BPHS facility, or elsewhere (chi^2^: 17.090; p = .0002), with significantly more care-seeking from CHWs and in BPHS facilities for fever than for ARI (p = .0001) or diarrhea (p = .0357), but no statistically significant difference between ARI and diarrhea. Private clinics or pharmacies were the second most frequent source (19%) and consulting a relative, 17%. Traditional healers accounted for a very small proportion (2%) of the cases in which the family sought health care for a sick child. No statistically significant association emerged between type of illness and different types of non-BPHS sources of care.

Of the 62%, or 186, children who were ill and were taken to a CHW or BPHS facility for treatment, most of those (59%) were taken to a clinic (Table 1). The remaining children were nearly evenly divided, with 22% taken to see the CHW at the health post and the other 19% taken to the hospital for care. There was no statistically significant association between the type of illness and the type of BPHS facility first consulted for care.

### Referral patterns for sick children

Of the 302 sick children about whom advice was sought outside the home, the first-line health care provider referred 33% (99) of them to another health care provider (Table 2). ARI accounts for nearly 60% of the cases referred to a higher level by the first health care provider seen. But the differences in the percentages of referrals by first health care providers to a higher-level provider by health problem were minimal—35.5%, 33.9%, and 26.3% for ARI, diarrhea, and fever, respectively (Table 2)—and are also not statistically significant.

**Table 2:** Number of sick children referred, illness for which referred and referral destination, by first source of care

**First source of care**	**Sick children**										
	**Seen**	**Referred**			**Not Referred**		**Referred**								**Not Referred**		**P value**^**1**^
CHW	41	29	71%		12	29%	29	71%	12	29%	
Clinic	110	23	21%		87	79%	30	21%	115	79%	<.0001
Hospital	35	7	20%		28	80%					
Private clinic / pharmacy	58	6	10%		52	90%	39	34%	77	66%	<.0001
Traditional healer	6	3	50%		3	50%					
Relative/friend	52	30	58%		22	42%					
Total	302	99	33%		203	67%	^!.^ Comparing CHWs with others				
First source of care	Illness for which referred										
	Seen	ARI			Diarrhea		Fever				
CHW	41	24	83%		3	10%	2	7%			
Clinic	110	14	61%		4	17%	5	22%			
Hospital	35	5	71%		1	14%	1	14%			
Private clinic / pharmacy	58	1	25%		1	25%	2	50%			
Traditional healer	6	1	33%		1	33%	1	33%			
Relative/friend	52	13	43%		8	27%	10	33%			
Total	302	59	60%		19	19%	21	21%			
Referral destination											
First source of care	CHW		Clinic		Hospital		Private pharmacy		Other		
CHW	1	3%	27	93%	1	3%	0	0%	0	0%	
Clinic	1	4%	10	44%	7	30%	5	22%	0	0%	
Hospital	0	0%	1	14%	6	86%	0	0%	0	0%	
Private clinic / pharmacy	0	0%	1	25%	3	75%	0	0%	0	0%	
Traditional healer	0	0%	1	33%	1	33%	0	0%	1	33%	
Relative/friend	1	3%	19	61%	9	29%	1	3%	1	3%	
Total	3	3%	59	60%	28	28%	7	7%	2	2%	

The large majority of children brought first to a CHW, friend, relative, or traditional healer were referred to another care provider. Only about 20% of children brought first to a clinic or hospital were referred elsewhere. Few of the children brought to a pharmacy or a private practitioner were referred elsewhere. The difference in referral patterns is statistically significant for the association between source of the first care being a CHW rather than a BPHS facility or other non-BPHS provider (p < .0001). The result is similar if we combine “CHW” and “Friend, relative” into one category, and compare with BPHS facilities and other non-BPHS providers. There is no statistically significant association between referral pattern and the first source of care being a BPHS facility or a non-BPHS source of care.

### Specificity of referral advice

#### Recommended first referral site

When we examined where sick children were referred (Table 2), a stepwise pattern respecting the different levels of care emerged. In other words, CHWs referred 93% of referred children to a clinic. Likewise, relatives or friends referred sick children primarily to clinics or hospitals. Those initially seen at a clinic were usually referred to another clinic or a hospital. Those initially seen at a hospital were referred only to another hospital, as we would expect.

#### Urgency of referral and referral slips

The urgency of the referral or the recommended delay in referral (Table 3) varied by the initial health care provider (Table 3). More than half of the referred cases were told to seek referral within 24 hours (immediately or same day), and another quarter were told to seek care at a higher level if the child’s condition worsened. In over 20% of the cases, no guidance was given about when caretakers should seek care at a higher level, or the parent could not recall if it was provided. The difference in proportion of children seen by CHWs getting no guidance (10%) is statistically significantly different from the proportion seen by BPHS facilities getting no guidance (28%, p = .0210) but not when comparing these proportions between CHWs and other sources of care.

**Table 3:** Urgency of referral, use of referral slips and compliance with referral, by first source of care

**First source of care**	**Urgency of referral**										
	**Immediately**		**Same day**		**If worse**		**Not specified**		**No recall**		**p value**^**1**^
CHW	6	21%	11	38%	9	31%	2	7%	1	3%	
Clinic	2	9%	5	22%	7	30%	8	35%	1	4%	.0210
Hospital	5	71%	1	14%	0	0%	1	14%	0	0%	
Private clinic/ pharmacy	4	67%	1	17%	1	17%	0	0%	0	0%	.5072
Traditional healer	1	33%	0	0%	1	33%	1	33%	0	0%	
Relative/friend	11	36%	6	19%	7	23%	5	16%	2	7%	
Total	29	29%	24	24%	25	25%	17	17%	4	4%	
			1. Comparing CHW with BPHS facilities and All others combined								
First source of care				Referral slip given											
	Yes		No		p value^2^	p value^3^	2. Comparing CHW with BPHS facilities and with all others combined								
CHW	21	72%	8	28%			3. Comparing BPHS facilities with all others combined								
Clinic	5	22%	18	78%	.0040										
Hospital	5	71%	2	29%											
Private clinic/ pharmacy	3	50%	3	50%	<.0001	.0439									
Traditional healer	1	33%	2	67%											
Relative/friend	1	3%	30	97%											
Total	36	36%	63	63%											
First source of care				Complied with referral											
	Yes		No		1^st^ Source	Yes		p value							
CHW	23	79%	6	21%	Community level	53	84%								
Clinic	15	65%	8	35%											
Hospital	6	86%	1	14%	Public or private facility	22	61%	.0146							
Private clinic/ pharmacy	1	17%	5	83%											
Traditional healer	3	100%	0	0%											
Relative/friend	27	87%	4	13%											
Total	75	76%	24	24%	Total	75	76%								

The data from CHWs and hospitals showed the highest percentages of referred children who were provided with referral slips. More than three-quarters of sick children referred from clinics to a higher level were sent without a referral slip. There is a statistically significant association between first source of care and receiving a referral slip, with CHWs giving more referral slips than BPHS facilities (p = .0040) and more than other non-BPHS sources of care (p < .0001), and BPHS facilities giving more referral slips that non-BHS sources of care (p = .0439).

There is a statistically significant relationship between the urgency of care and receiving a referral slip, with the more urgent getting more referral slips (chi^2^: 8.462, p = .0132), in particular when comparing referral within 24 hours (immediate and same day) with non-specified and non-recalled advice (p = .0135)

### Compliance with referral advice

The majority of caretakers complied with the advice to seek referral (Table 3): 76% of all those who received advice to go to a higher-level health care provider actually went. Those initially seen by CHWs, at hospitals, or by traditional healers complied with the referral advice to the greatest extent. A slightly smaller proportion of parents of sick children who first went to a clinic complied with the referral advice (65%). Almost 90% of those referred by a relative or friend complied with the referral advice, despite not receiving a referral slip. When we compare all children referred from the community level (CHW, friend/relative, traditional healer) with those referred from a health facility (BPHS facility, hospital, private clinic/pharmacy), there is significantly more compliance for those referred from the community level (p = .0146).

Although there seems to be a positive relationship between the urgency of referral advice and compliance (Table 4), the association is not statistically significant. There is no statistical association between the referral destination and compliance with referral.

**Table 4:** Whether referral slip was given, compliance with referral, and distance traveled to referral facility, by urgency of referral and referral destination

**Urgency of referral**	**Referral slip given**					**Complied with referral**				**Time travelled to referral facility**					
	**Yes**		**No**		**p value**^**1**^	**Yes**		**No**		**≤ 1 hour**		**>1 and ≤2 hours**		**>2hours**	
Immediately	13	46%	15	54%		23	82%	5	18%	13	57%	9	39%	1	4%
Same day	12	50%	12	50%		19	79%	5	21%	11	58%	6	32%	2	11%
If worse	7	27%	19	73%		18	69%	8	31%	11	61%	6	33%	1	6%
Not specified	3	18%	14	82%	.01235	13	77%	4	24%	3	23%	7	54%	3	23%
No recall	1	25%	3	75%		2	50%	2	50%	1	50%	1	50%	0	0%
Total	36	36%	63	63%		75	76%	24	24%	39	52%	29	39%	7	9%
	^1^ Between <24 hours and unspecific														
Referral destination	Yes		No		Yes		No		≤ 1 hour		>1 and ≤2 hours		>2 hours		
CHW	1		33%	2	67%	3	100%	0	0%	3	100%	0	0%	0	0%
Clinic	23		39%	36	61%	48	81%	11	19%	25	52%	19	40%	4	8%
Hospital	11		39%	17	61%	20	71%	8	29%	9	45%	8	40%	3	15%
Private pharmacy	1		14%	6	86%	3	43%	4	57%	2	67%	1	33%	0	0%
Other	0		0%	2	100%	1	50%	1	50%	0	0%	1	100%	0	0%
Total	36		36%	63	63%	75	76%	24	24%	39	52%	29	39%	7	9%

Our data confirm that having a referral slip encouraged parents or caretakers to take sick children to the next level of care. Nearly 90% of those with referral slips complied with the referral advice and sought care, as compared with only 50% of those who did not receive a referral slip, and that association in statistically significant (p = .0277).

### Potential barriers to access to referral health care provider

Compliance with referrals depends not only on sound decisions by the family to seek care and on referral decisions by the first health care provider seen, but also on the family’s decision to follow through on the advice of the referring provider to seek further care [17]. The family’s decision to go to the higher-level health care provider is influenced by many factors influencing access to the higher level services, including the distance to the facility, transport available, costs associated with travel, and satisfaction with the higher-level health care provider based on previous experiences.

#### Distance to referral health care provider

More than half of the 75 who went to the indicated referral site travelled 1 hour or less, and more than 90% travelled 2 hours or less, with little difference for urgency of referral, first care site, or referral destination (Table 4). None of these differences show a statistically significant association.

#### Means and costs of getting to referral health care facility

Of the 75 children that were brought to the referral site, more than 50% walked, and less than 10% used a vehicle provided by the health facility. There is no statistically significant association between urgency of referral and transportation means, but there is a statistically significant association between the first source of care and means of transportation: 71% of those who went to BPHS facility (clinic or hospital) used a vehicle compared to 29% for all other first sources of care (p = .0073).

The majority (63%) of all patients who went to the indicated referral site did not pay anything for transport or travel, largely because more them half of them (40 of 75) walked. There is no statistically significant association between first source of care and paying or not paying for transport, nor between using a vehicle provided by the facility and paying or not paying for transport. A larger proportion of those that went to hospitals (60%) paid than of those who went to clinics (25%), and that association is statistically significant (p = .0111).

Of all those who paid something (28 of 75, or 37%), one-half paid more than 100 Afs (US$2.00) at the time of the study. Most frequently, patients paid for vehicle transport that was not provided by the referring health facility, and there is a statistically significant association between paying more than 100 Afs and using a vehicle not provided by the first care facility (p = .0084). The numbers are too small to calculate confidence intervals, however.

These summary data on transportation costs do mask wide variations (Table 5). If we disregard the extreme outlying value of 5,000 Afs paid to reach one CHW, on average 564 Afs was paid, and more was paid on average to get to hospitals (661 Afs) than to clinics (185 Afs).

**Table 5:** Referral travel costs by referral site

**Referred to**	**Transport cost in Afs.**		
	**Minimum**	**Maximum**	**Mean**
CHW	5,000	5,000	5,000
Clinic	10	500	185
Hospital	40	2,000	661
Pharmacy	20	50	35
Other	200	200	200
Total	10	5,000	564

#### Patient satisfaction influencing compliance with referral

Only 2 of 75 parents said that they did not want to go back to the health facility to which they were referred. The reasons cited for dissatisfaction with the facility was distance in one case and disrespectful behavior by the staff toward the child’s caretakers in the other case. The transport cost to get to hospitals was reported to be too high, although the amount paid was 300 Afs (US$6.00), which was below the average paid.

Of the 99 children who were referred to another facility or health worker, 24 parents and caretakers (24%) did not comply with the referral advice for the sick child. Table 6 lists the reasons mentioned by caretakers for not going to the recommended referral site. More than 50% list reasons related to transportation (weather, road blocked, too far, transportation costs). Family-related reasons make up 21% (nobody to take care of other children, nobody to take the child, no permission to go). Perceived poor quality of care at the referral facility (unskilled staff, no medicine) was given as a reason in 10% of the cases.

**Table 6:** Reasons for not going to recommended referral site

**Reason for not attending referral facility**	**Recommended referral site**					
	**Clinic**	**Hospital**	**Pharmacy**	**Other**	**Total**	
Weather/road blocked	6	1			7 (24%)
Distance	3	1	1	1	6 (21%)
No one to care for siblings	2	3			5 (17%)
Transportation cost	1	2			3 (10%)
Staff not skilled		1	1		2 (7%)
No medicine at the facility	1				1 (3%)
Did not have permission to go		1			1 (3%)
Other	1	1	3		5 (17%)
Total not following referral advice	13 (45%)	10 (35%)	5 (17%)	1 (3%)	29^a^

^a^ Since some respondents gave 2 answers, the responses totaled 29 for 24 people interviewed.

Assuming that those who did not go the referral facility only because of reasons related to transport would go if free or affordable transport were available, the percentage that would still not go would drop to 13%, a difference that is not statistically significant. If we assume, however, that all those who mentioned a reason related to transport (weather, road blocked, too far, transportation costs) would go if free or affordable transport were available, the percentage that would still not go would drop to 8%, a difference that becomes statistically significant (p = .0033).

Of the 24 who did not go to the referral health facility, 6 (25%) stated that they chose an alternative: 1 went to a CHW, 2 to clinics, 2 to private clinics instead of hospitals, and 1 to a pharmacy.

## Discussion

### Care-seeking behavior for sick children

For proper referrals of young children, the first requirement is a parent or caretaker seeking the initial consultation. This did not appear to be a major issue in Afghanistan, since parents or caretakers of the sick child sought care from a health care provider in more than 60% of the episodes of illness. The influence of elders, including mothers-in-law and grandparents, in traditional Afghan society may explain why parents complied with relatives’ and friends’ recommendations to seek care for sick children almost 90% of the time.

Parents chose government-provided health services in 62% of the cases, most often (81%) from a primary care health worker (a CHW at a basic or comprehensive health center), whereas hospitals represented the first source of care in only 19% of the cases (Table 1). This can be explained because most of the families of the selected sick children live in rural districts, and the primary care facilities are closest to the home. But it is also encouraging that the data do not show a strong tendency to bypass the first level of primary care.

Caretakers of children with fever sought help outside the home in 74% of the cases, significantly more than those of children with diarrhea (51%, p = .0005) or ARI (61%, p = .0131). Only 43% of the fever cases were brought to a BPHS facility, significantly less than diarrhea cases (63%, p = .0357) or ARI cases (71%, p < .0001). A household survey in 1977 found that child mortality was associated with *jinns* (fever), ARI, and diarrhea; however, diarrhea and ARI but not *jinns* (fever) were mentioned as treatable health problems. Persistence of the perception that fever may kill children, but is not necessarily treatable by health workers, may partly explain the present findings [18]. The type of illness was not associated with significantly different care-seeking between BPHS facilities or between non-BPHS sources of care.

### Health workers’ actions

A second requirement for a good referral system is that the health care provider at the first place where care is sought recognizes severe conditions in ill children and takes prompt action to refer the child to a higher-level health facility. Of the 302 children who sought care from a health care provider, one-third (99) were referred to a higher-level health care provider or facility. The predominant condition for which there was a referral was ARI, at 60%, while the remaining cases were almost evenly divided between diarrhea and fever. There is no statistically significant association between type of illness and referral to another source of care. These proportions appear to be consistent with general morbidity patterns of diseases in Afghanistan.

CHWs and relatives or friends referred more than half of the children seen. The difference in proportion of sick children referred by CHWs, BPHS facilities, and other sources of care is statistically significant (chi^2^: 36.571, p < .0001). CHWs, who have limited training and are not trained in emergency stabilization of patients, may have a tendency to over-refer. Since ARI was the most common condition for referrals, on one hand, it is encouraging that ARI cases are expected to be referred without delay, because if children are not treated promptly and appropriately, ARI can easily develop into severe, life-threatening pneumonia. The referral rates by CHWs seem very high, on the other hand. This is a concern, since CHWs are trained and expected to treat uncomplicated pneumonia without referral.

### Urgency of care and use of referral slips

Because a key element of IMCI is immediate referral of serious cases to a higher-level health care provider or facility, IMCI guidelines instruct health workers to give a referral note to the parent or caretaker of the child as well as information and counseling about the urgency of the referral, location of the referral facility, and advice about any barriers that would prevent the parent or caretaker from taking the child to the referral facility as soon as possible.

It seems that an adequate number of children were referred to a higher-level health facility and that the referrals adequately accounted for the level of urgency, since only 21% of referrals did not specify how quickly the child needed to see the higher-level health care provider (or the family member did not recall if that was specified). So nearly 80% were advised to seek referral care immediately, on the same day, or if the child’s condition worsened. CHWs are significantly more specific in their advice than BPHS facilities (p = .0210).

Only 36% of referrals used a referral slip (Table 3), however. This is problematic, since providing a referral slip to the parent or caretaker of a very ill child has been shown to be directly related to the degree of compliance with the referral. (Kalter, 2003). As could be expected, fewer referral slips are given when families are referred by sources of care outside the public health system (13%), which is significantly fewer than at BPHS facilities (33%, p = .0439) and by CHWs (73%, p < .0001). CHWs do significantly better than BPHS facilities (p = .0040). While the poor use of referral slips in BPHS facilities is cause for concern, the higher use of referral slips by CHWs is encouraging.

A positive finding was that referral slips were provided in the highest proportion of cases where the referral was deemed urgent (“immediately” or “same day”). In particular, a significantly higher proportion of referrals within 24 hours receive a referral slip (69%) compared to unspecified referrals (36%, p = .0113). Although we did not ask directly about counseling, it appears that there was minimal to no counseling of parents or caretakers about the child’s condition and the reasons for the urgent referral.

### Compliance with referral advice and referral constraints

Compliance was generally good, with over 75% actually going to the higher-level health care provider or facility when referred. Compliance with the referral seems independent of the type of illness, the destination of referral, or whether the urgency of care was specified. One factor that significantly influenced compliance with referral was whether a referral slip was provided to the caretaker (89% compared to 50% when there was no referral slip, p = .0277), This finding is in line with findings in other countries and studies. Another factor influencing compliance was whether the referral was advised by somebody in the community (CHW, friend/relative, traditional healer) versus somebody in a health service outlet (BPHS facility, private clinic/pharmacy): 81% compared to 61% (p = .0146).This may be explained by the traditional respect given to decision-makers in the community, and possibly because barriers for compliance may be less important between the community and first-level facilities than between the community and second level facilities.

There were some barriers to complying with the referral advice the first-level health care provider gave, but these were not as great as some studies have shown in other countries. The distances were not excessive for rural populations, with less than 10% of the referrals being to health facilities that were more than 2 hours away. With 90% of referrals being within 2 hours or less, vehicle use did not appear to be as significant as we expected: vehicles were used in just over 40% of the cases, while walking or use of an animal accounted for 60% of the transportation usage by referred patients. Use of a vehicle by those seen by BPHS facilities was significantly higher than by those seen elsewhere (71% compared to 29%, p = .0073) This relatively low use of vehicles also resulted in the costs of transport being generally moderate (except for hospitals) and thus not a barrier to access to the referral facility in a significant number of referred cases.

The lack of free or inexpensive motorized transportation is often given as a major reason why patients do not follow referral advice. Kowalewski et al. [19] found that financial and geographical (transport) difficulties represented well-known barriers to at-risk mothers’ following referral advice. Costly transportation was clearly identified as a barrier affecting compliance with referrals in rural Tanzania [20].

Comparing the cost of a loaf of *naan*, a flat bread that is a staple of Afghans’ diet, 6 Afs. at the time of the survey, with the average cost of transport (564), then transport costs almost 100 times more than one loaf, and about 16 times what an average household would spend on *naan* a day. We should consider also that most of the vehicles were private vehicles, for which the large majority (86%) paid, and also that all those that paid more than 100 Afs for transport paid for private vehicles.

Assuming that those who did not go the referral facility only because of reasons related to transport (weather, road blocked, too far, transportation costs) would go if free or affordable transport were available, the percentage that would still not go would drop from 24% to 13%, a difference not statistically significant. However, if we would assume that all those who mentioned a reason related to transport would go if free or affordable transport were available, the percentage that would still not go would drop to 8%, a difference that becomes statistically significant (p = .0033). This may further indicate that, at least for some, the cost of transport or absence of affordable transport might be a barrier also in Afghanistan. The numbers are, however, very small, and the questionnaire did not really investigate this in more detail. Definitely this issue deserves a more formal assessment with a larger sample before drawing conclusions.

Our findings about patient satisfaction were positive, in that the majority of referred patient families would be willing to use the referred facility again based on their experience. This seems different from earlier studies of access for the poor living in rural areas, which have shown that poor treatment of patients represents a large barrier in use of health facilities [21]. In Afghanistan positive patient perceptions may have been aided because the quality of health care for the rural poor in Afghanistan has improved due to intensive support from numerous donors in rebuilding the health system, including training and supervision of health workers.

## Conclusions

Appropriate and timely referral of sick children is a cornerstone of IMCI. This study confirms that in Afghanistan referral patterns seem to reflect disease patterns as well as the perception of communities about what conditions are best treated with modern medicine. Compliance with referral recommendations is higher from the community to the health facilities than from one facility to another one. The study also confirms the importance of a referral slip to improve compliance with referral. Training health workers in counseling of caretakers holds promise to change health care-seeking behavior and increase the number of successful referrals but falls outside the scope of this study.

The study is less clear on the importance of financial barriers linked to transportation, which warrants further investigation; in particular if a policy decision on where ambulances may improve compliance needs to be made.

## Competing interests

The authors declare that they have no financial nor non-financial competing interests.

## Authors’ contributions

WN and PI helped conceptualize the study, development of the survey instrument, compilation of the data, statistical analysis of the data and writing of the manuscript. RW helped in conceptualizing and designing the survey instrument, did the literature search, wrote up the literature search and reviewed and commented on the document. FM helped design the survey instrument, helped train and supervises the data collectors, participated in reviewing the data results and commenting on the results. All authors read and approved the final manuscript.

## Pre-publication history

The pre-publication history for this paper can be accessed here:

http://www.biomedcentral.com/1471-2431/12/46/prepub
